# Early Reactive A1 Astrocytes Induction by the Neurotoxin 3-Nitropropionic Acid in Rat Brain

**DOI:** 10.3390/ijms21103609

**Published:** 2020-05-20

**Authors:** Carmen Lopez-Sanchez, Virginio Garcia-Martinez, Joana Poejo, Virginio Garcia-Lopez, Jairo Salazar, Carlos Gutierrez-Merino

**Affiliations:** 1 Instituto de Biomarcadores de Patologias Moleculares, Universidad de Extremadura, 06006 Badajoz, Spain; virginio@unex.es (V.G.-M.); joanapoejo86@gmail.com (J.P.); garcialopez@unex.es (V.G.-L.); jairochemsalazar@gmail.com (J.S.); carlosgm@unex.es (C.G.-M.); 2 Departamento de Anatomia y Embriologia Humana, Facultad de Medicina, Universidad de Extremadura, 06006 Badajoz, Spain; 3 Departamento de Enfermeria, Facultad de Medicina, Universidad de Extremadura, 06006 Badajoz, Spain; 4 Departamento de Quimica, Universidad Nacional Autonoma de Nicaragua-Leon, Leon 21000, Nicaragua; 5 Departamento de Bioquimica y Biología Molecular y Genetica, Facultad de Ciencias, Universidad de Extremadura, 06006 Badajoz, Spain

**Keywords:** 3-nitropropionic acid, rat brain, A1 astrocytes, complement component 3, cytokines IL-1α, TNF-α and C1

## Abstract

3-Nitropropionic acid (NPA) administration to rodents produces degeneration of the *striatum*, accompanied by neurological disturbances that mimic Huntington’s disease (HD) motor neurological dysfunctions. It has been shown that inflammation mediates NPA-induced brain degeneration, and activated microglia secreting cytokines interleukin-1α (IL-1α) and tumor necrosis factor α (TNFα) can induce a specific type of reactive neurotoxic astrocytes, named A1, which have been detected in post-mortem brain samples of Huntington’s, Alzheimer’s, and Parkinson’s diseases. In this work we used an experimental model based on the intraperitoneal (i.p.) administration of NPA to adult Wistar rats at doses that can elicit extensive brain degeneration, and brain samples were taken before and after extensive brain damage monitored using 2,3,5-triphenyltetrazolium chloride (TTC) staining. Western blots and immunohistochemistry of brain slices show that i.p. NPA injections elicit significant increase in the expression levels of C3α subunit, a marker of generation of neurotoxic A1 astrocytes, and of cytokines IL-1α, TNFα, and C1q within the *striatum*, *hippocampus,* and *cerebellum* before the appearance of the HD-related neurological dysfunctions and neuronal death induced by NPA. Noteworthy, NPA administration primarily induces the generation of A1 astrocytes in the more recent phylogenetic area of the rat *cerebellum*. We conclude that the activation of complement C3 protein in the brain from Wistar rats is an early event in NPA-induced brain neurodegeneration.

## 1. Introduction

3-Nitropropionic acid (NPA) is a natural toxin produced by some fungi and plants [1,2,3,4]. Systemic NPA administration to rodents and non-human primates produces degeneration of the *striatum*, accompanied by neurological disturbances that mimic Huntington’s disease (HD) motor neurological dysfunctions. More precisely, systemic NPA administration produces a selective bilateral degeneration in the dorso-lateral region of the *striatum* [5,6,7,8,9,10], resembling severe damage observed in the caudate and dorso-lateral *putamen* of human brains affected by HD [11]. Moreover, preferential degeneration of the medium-sized GABAergic spiny striatal neurons has been reported after NPA systemic administration, alterations also observed in HD *striatum* [5,7]. However, the pre-motor symptomatic stages of HD are commonly characterized by cognitive issues, including executive dysfunction, visuospatial deficits, perceptual deficits, memory loss, and difficulty in learning new skills [12,13]. It has been proved that systemic NPA administration to rodents also produces memory impairment [14,15], and significant metabolic alterations, not only in the *striatum* and vicinal cortical areas, but also in the *hippocampus* [15]. Furthermore, it has been shown that NPA administration to rats also elicits significant changes both to metabolism and neurotransmitters in the *cerebellum* [16,17]. Noteworthy, cerebellar *cortex* damage with extensive Purkinje cells loss has been reported recently in post-mortem samples of HD cases [18].

Mitochondrial functional alterations as well as associated generation of reactive oxygen species (ROS), that activate cell death pathways, have been demonstrated to play a major role in NPA neurotoxicity [19,20,21]. Additionally, both processes have been implicated in HD [22]. NPA is a suicide inhibitor of succinate dehydrogenase and causes rapid loss of ATP in neurons in vitro [19,23]. Depletion of neuronal ATP leads to a sustained rise in cytosolic calcium due to the large consumption of ATP to restore plasma membrane potential after repetitive synaptic activity in brain neurons. This potentiates excitatory neurotransmitter secretion and, eventually, neuronal death through calpains activation. Moreover, by means of cellular and animal experimental models, NPA has been shown to promote excitotoxic neuronal death, mediated by the excitatory neurotransmitters l-glutamate and dopamine [17,24,25], and by calpains activation [19,26,27]. Furthermore, it has been shown that NPA produces indirect excitotoxic damage to the *striatum* [28], making it unlikely that NPA-induced selective damage of neurons in specific regions of the brain can be solely related with the metabolic rate and density of receptors for l-glutamate and dopamine.

Activated microglia in the brain is a major source of ROS and nitric oxide in the brain and this can also impair the mitochondrial respiratory chain function [29], and in a previous publication [10] we have shown a large decrease of reduced glutathione in parallel with a large increase of protein nitrotyrosines in NPA-induced degeneration of the striatum of adult Wistar rats brain. This is consistent with activation of neuroinflammatory microglia by NPA administration reported by others [30,31,32] and strongly suggests that inflammation mediates NPA-induced brain degeneration. Noticeably, non-invasive imaging of the human brain has revealed significant microglial activation both in the *striatum* and cortical areas in HD patients [33]. Indeed, it has been proposed that pro-inflammatory cytokines stimulate the development of neurodegenerative diseases—including Huntington´s, Alzheimer’s, and Parkinson’s—where there is a slow and progressive damage of cerebral cortical areas: *substantia nigra*, *striatum*, and *hippocampus* [34]. Pro-inflammatory cytokines may potentiate oxidative stress-induced cell death through enhanced production of ROS and nitric oxide and stimulation of l-glutamate release to the extracellular space [35]. In addition, the release of endogenous cell molecules during brain degeneration (Damage Associated Molecular Patterns or DAMPs) can elicit further microglial activation, establishing a positive feedback loop in those brain areas undergoing a more extensive degeneration, such as the *striatum* and vicinal somatomotor *cortex* [36].

However, the molecular mechanisms through which brain inflammation can selectively produce cell death in specific neuronal structures remain unclear. Astrogliosis and loss of astrocytes have been noticed in the brain *striatum* in rats treated with NPA [28,31,32,37]. Recently, it has been shown that neuroinflammatory microglia activation can induce the formation of a specific type of reactive neurotoxic astrocytes, named A1, through the secretion of specific cytokines interleukin-1α (IL-1α), tumor necrosis factor α (TNFα) and complement component 1q (C1q), and that reactive A1 astrocytes are abundant in post-mortem tissue of HD patients [38]. Moreover, it has been noted that complement component 3 (C3) is a highly upregulated gene in A1 astrocytes, while it is not expressed by ischemic reactive astrocytes, named A2 [38]. Thus, C3 expression can be used to highlight the induction of reactive neurotoxic A1 astrocytes in neurodegenerative disorders.

In this work we used an experimental model based on the intraperitoneal administration of NPA to rats at doses that can elicit extensive brain degeneration, and brain samples were taken before and after extensive brain damage can be noticed. We show that reactive A1 astrocytes, expressing the activated C3α fragment, are induced by NPA treatment in the *striatum*, as well as in highly relevant neuronal structures inside both the *hippocampus* and the *cerebellum*. Consistent with this finding, an increase of the levels of pro-inflammatory cytokines IL-1α, TNFα, and C1q was also detected in these areas of the brain of rats treated with NPA.

## 2. Results

In this work we designed an experimental NPA-treatment to induce progressive brain degeneration to investigate early biomarkers before the appearance of neurological defects and neuronal death.

### 2.1. The Increase of Activated Complement C3 Protein, a Reactive A1 Astrocyte Marker, Precedes Significant NPA-Induced Brain degeneration

**Figure 1:**
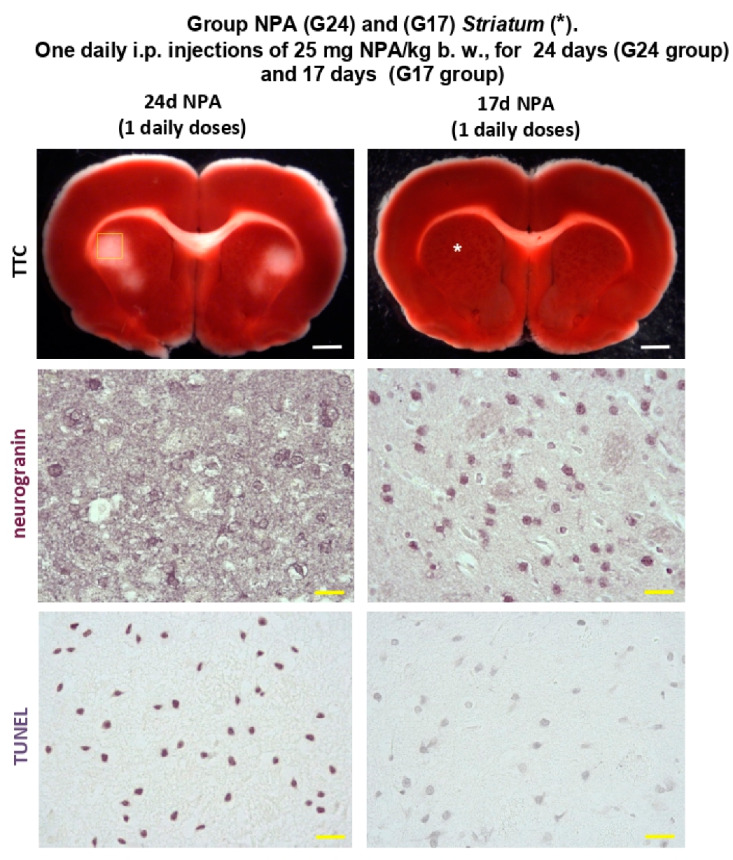
Rats of the G24 group show degeneration of the brain *striatum* (*) with respect to rats of the G17 group. Representative coronal sections stained with 2,3,5-triphenyltetrazolium chloride (TTC), neurogranin, and TUNEL, as indicated in Section 4. Treatment with one daily i.p. injection of 25 mg 3-nitropropionic acid (NPA)/kg b. w. for 24 days (G24 group) shows an initial unstained area of the *striatum* after TTC staining with respect to rats of the G17 group. Yellow square mark indicates the selected TTC unstained area neurogranin and TUNEL staining. Comparative neurogranin immunolabeling reveals a loss of neuronal somas in the *striatum* of rats of the G24 group with respect to rats of the G17 group. Comparative TUNEL staining shows neuronal cell death in the *striatum* (*) of rats of the G24 group. White scale bars: 2 mm. Yellow scale bars: 25 µm.

Adult Wistar rats treated i.p. with a daily dose of 25 mg NPA/kg body weight up to 24 days (G24) already showed neurological defects based on increased dystonic movements of hind limbs and an abnormal gait, characterized by a wobbly gait and padding. These neurological symptoms are characteristics of this HD experimental model, as we previously described [10]. Furthermore, 2,3,5-triphenyltetrazolium chloride (TTC) staining of rat brain slices present evident damage of the *striatum* (Figure 1). Additionally, neuronal loss detected by means of neurogranin immunostaining and apoptotic cells observed with TUNEL staining reveal the neuronal damage related with the pathological events above described (Figure 1).

**Figure 2:**
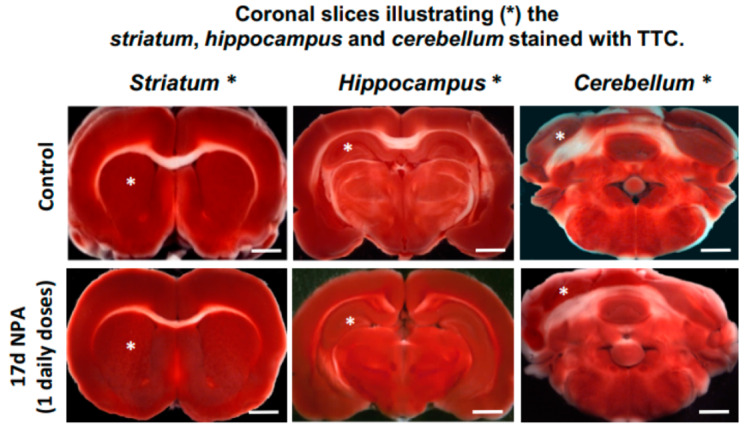
Representative fresh brain 1.5 mm thick coronal slices illustrating the *striatum*, *hippocampus,* and *cerebellum* (respectively marked with white asterisk) stained with TTC. Treatment with one daily i.p. injection of 25 mg NPA/kg b. w., for 17 days (G17 group) show no differences with TTC staining of control rats. Scale bars: 2 mm.

Interestingly, rats treated i.p. with a daily dose of 25 mg NPA/kg body weight up to 17 days (G17) did not show significant sensorial or motor neurological dysfunctions yet, although after 17 doses some rats became hypoactive, but keeping a normal posture and gait. Indeed, G17 staining of coronal rat brain sections showed no differences with TTC staining of untreated control rats (Figure 2). The lack of noticeable unstained white areas in the *striatum*, *hippocampus,* and *cerebellum* of rats treated i.p. with one daily dose of 25 mg NPA/kg, for 17 days, pointed out that this treatment still did not produce observable degeneration of these brain areas. This result is supported by the neuronal soma labeling with neurogranin and no evident apoptotic cells with TUNEL in the *striatum* (Figure 1).

**Figure 3:**
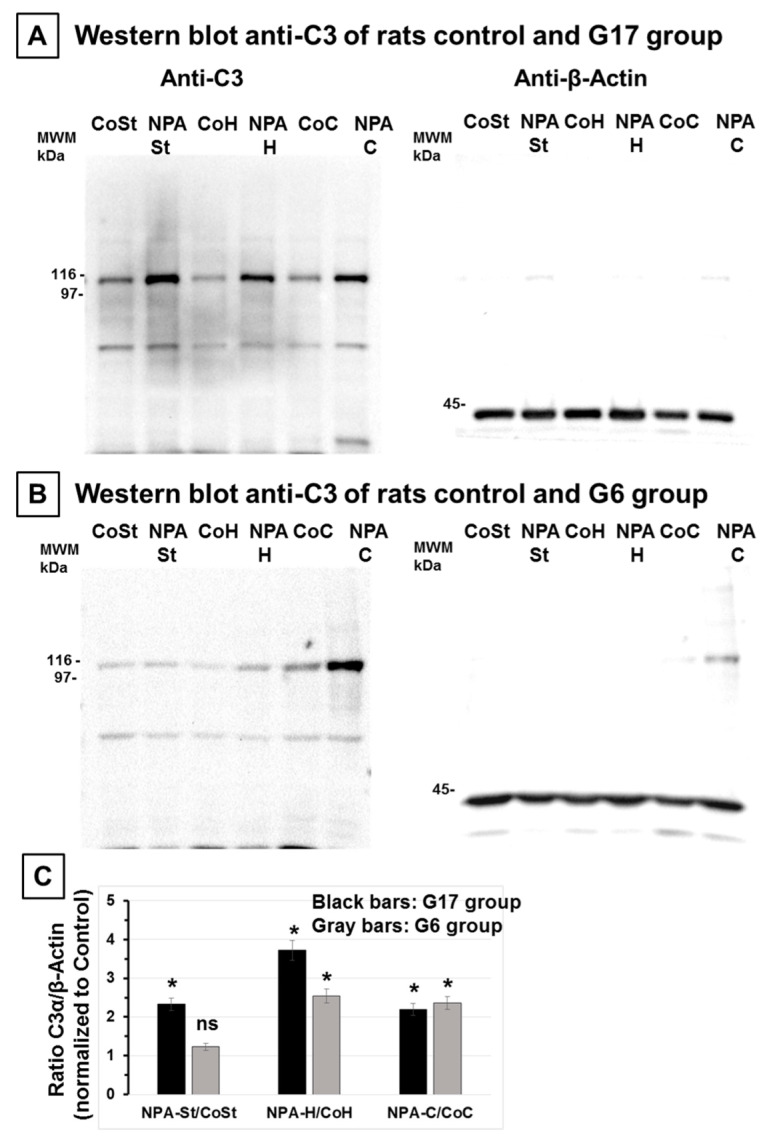
Rats of the G17 group show a large increase of the C3α proteolytic fragment in the *striatum*, *hippocampus,* and *cerebellum* with respect to rats of the control group, while the C3α proteolytic fragment only significantly increases in the *hippocampus* and *cerebellum* in the rats of the G6 group. Representative Western blots of C3 and β-actin of *striatum* (St), *hippocampus* (H), and *cerebellum* (C) homogenates of rats of control (Co) and G17 group (NPA rats) (**A**) and of control (Co) and G6 group (NPA rats) (**B**). After acquisition of images of the Western blot with anti-C3, the polyvinylidene difluoride (PVDF) membrane was stripped and processed for the Western blot of anti-β-actin, as indicated in Section 4. The molecular weights of the protein markers (MWM) closer to the target proteins (C3α proteolytic fragment and β-actin) are indicated on the left-hand side. (**C**) Plot of the ratio of (C3α/β-actin) in *striatum*, *hippocampus,* and *cerebellum* homogenates of rats of G17 (black bars) and G6 (gray bars) groups relative to control rats. The results shown are the average ± s.e. of triplicate experiments. (*) *p* < 0.05 with respect to control rats; ns, statistically non-significant difference with respect to control rats.

On these grounds, we selected rats subjected to 17 days treatment (G17) for an experimental assessment of brain biomarkers at early stages of the NPA-induced neurotoxic process (Figure 3). Western blots reveal that proteolytic fragment C3α levels in the *striatum*, *hippocampus,* and *cerebellum* increased more than two-fold with respect to control untreated rats, namely 2.33 ± 0.17, 3.7 ± 0.25, and 2.2 ± 0.15-fold, respectively (Figure 3A,C). Notably, it has been shown that reactive A1 astrocytes are also produced upon neuronal axotomy [38], which can account for the low levels of C3α detected in these brain regions in control untreated rats. This result pointed out that reactive A1 astrocytes induced by this NPA treatment can be seen before significant NPA-induced brain damage is detected through TTC, neurogranin, and TUNEL staining, and precedes NPA-induced neurological dysfunctions.

Interestingly, brains of rats treated i.p. with a daily dose of 25 mg NPA/kg body weight for 6 days (G6) showed that a statistically significant increase of C3α levels with respect to control rats can only be observed in the *hippocampus* and *cerebellum*, i.e., an increase of 2.5 ± 0.2 and 2.4 ± 0.2-fold, respectively (Figure 3B). This fact indicates an early generation of reactive A1 astrocytes in these two brain regions.

### 2.2. The Increase of Proinflammatory Cytokines IL-1α and TNFα Also Precedes Significant NPA-Induced Brain Degeneration

**Figure 4:**
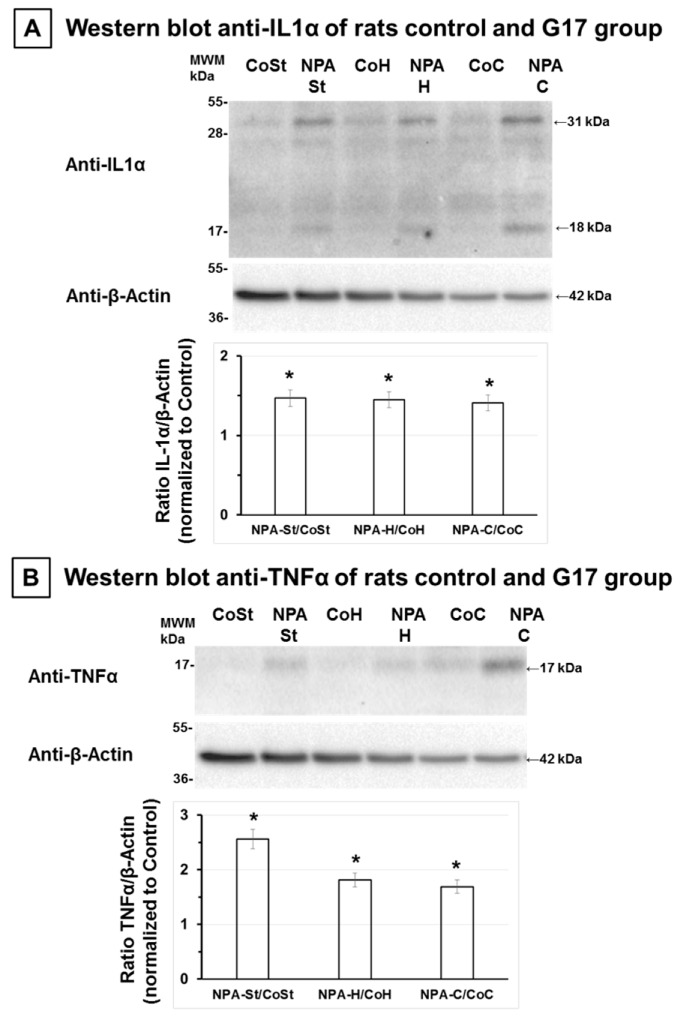
Rats of the G17 group show a significant increase of interleukin-1α (IL-1α) and tumor necrosis factor α (TNFα) in the *striatum*, *hippocampus,* and *cerebellum* with respect to rats of the control group. Representative Western blots of IL-1α and β-actin (**A**) and of TNFα and β-actin (**B**) of *striatum* (St), *hippocampus* (H), and *cerebellum* (C) homogenates of rats of control (Co) and G17 group (NPA rats). After acquisition of images of the Western blot with anti-IL-1α and TNFα, PVDF membranes were stripped and processed for the Western blot of anti-β-actin, as indicated in Section 4. The estimated molecular weights of the target proteins IL-1α, TNFα, and β-actin are indicated on the right hand side, and on the left hand side the molecular weights of the protein markers (MWM) closer to the target protein are included. Plots of the ratios of (IL-1α/β-actin) and (TNFα/ β-actin) in *striatum*, *hippocampus,* and *cerebellum* homogenates of rats of G17 group relative to control rats are also inserted, respectively. The results shown are the average ± s.e. of triplicate experiments. (*) *p* < 0.05 with respect to control rats.

Recent studies have shown that cytokines IL1-α and TNFα are specifically expressed in activated microglia induced by different brain injuries inducing reactive A1 astrocytes [38,39,40]. Therefore, by means of Western blotting, we measured by the expression level of these cytokines in the *striatum*, *hippocampus,* and *cerebellum* of rats treated i.p. with one daily dose of 25 mg NPA/kg body weight for 17 days (G17). Figure 4 shows that both IL1α and TNFα are increased in these brain areas with respect to control rats. The quantitative analysis yielded a similar increase of approximately 50% of the IL1α level in all brain areas under analysis (*striatum*, *hippocampus,* and *cerebellum*), while the increase of TNFα was higher in the *striatum* (160%) than in the *hippocampus* (80%) and *cerebellum* (70%).

### 2.3. Immunohistochemical Analysis of the Regionalization and Location of Complement C3 Protein Activation in the Striatum, Hippocampus, and Cerebellum

Our findings reveal a progressive increase in the activation of the complement component C3 protein from G6 through G17 groups. Since activation of C3 has earlier been identified as a reactive A1 astrocyte marker [38], we also used the astrocytic marker glial fibrillary acidic protein (GFAP).

**Figure 5:**
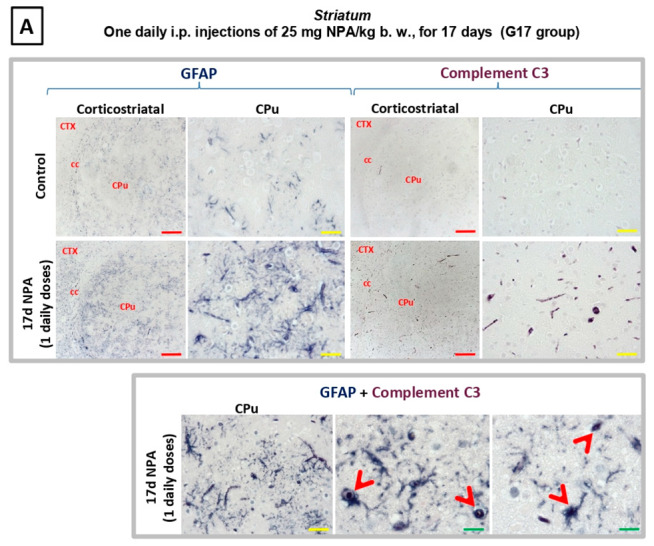

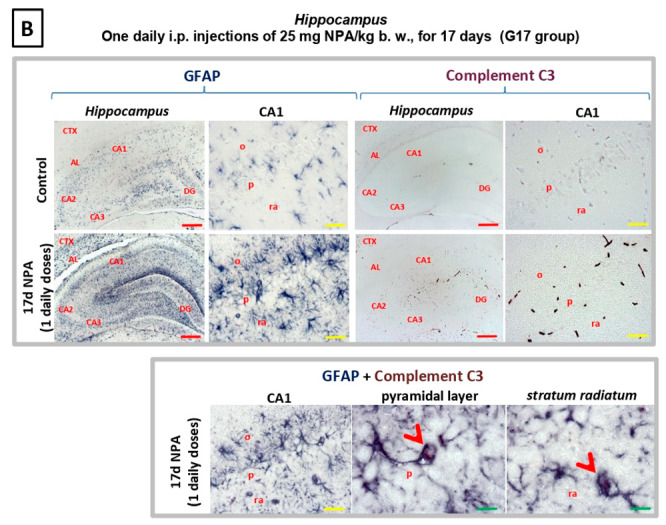
Rats of the G17 group (one daily i.p. injection of 25 mg NPA/kg b. w., for 17 days) show a significant increase of glial fibrillary acidic protein (GFAP) and Complement C3 in the *striatum* and *hippocampus* with respect to rats of the control group. Light micrographs of coronal sections after immunohistochemistry with anti-GFAP and anti-Complement C3 antibodies. (**A**) Comparative immunolabeling showing an increase in astrocytes expressing C3α and GFAP (upper panel), observed in the dorsolateral region of the *striatum* (*caudate putamen*) in experimental rats. Note that C3α and GFAP co-localize in the ameboid-shaped astrocytes (arrowhead), detected by using double immunohistochemistry (lower panel). (**B**) At the level of the *hippocampus* an increase both in C3α and GFAP is particularly significant within the pyramidal layer and *stratum radiatum* of CA1 area in Ammon’s horn (upper panel). Note that C3α and GFAP co-localize in astrocytes (indicated by red arrowheads), detected by using double immunohistochemistry (lower panel). In red letters: CPu: *striatum* (*caudate-putamen*); CTX: Cerebral *Cortex*; cc: *corpus callosum*; CA1: field CA1 of *hippocampus*; CA2: field CA2 of *hippocampus*; CA3: field CA3 of *hippocampus*; DG: dentate *gyrus*; AL: *alveus*; o: *stratum oriens*; ra: *stratum radiatum*; p: pyramidal layer. Red scale bars: 200 µm. Yellow scale bars: 25 µm. Green scale bars: 10 µm.

As illustrated in Figure 5, an increase in reactive A1 astrocytes—expressing C3α and GFAP—was observed in the dorsolateral region of the *striatum* (*caudate*-*putamen*) in NPA treated rats in experimental G17 group (Figure 5A). With respect to the *hippocampus*, we also observed an increase both in C3α and GFAP, particularly significant within the pyramidal layer and *stratum radiatum* of CA1 area in Ammon’s horn (Figure 5B). In addition, Figure 5 also highlights characteristic changes of the shape of astrocytes, and that C3α and GFAP co-localize in the ameboid-shaped reactive A1 astrocytes, detected by using double immunohistochemistry.

**Figure 6:**
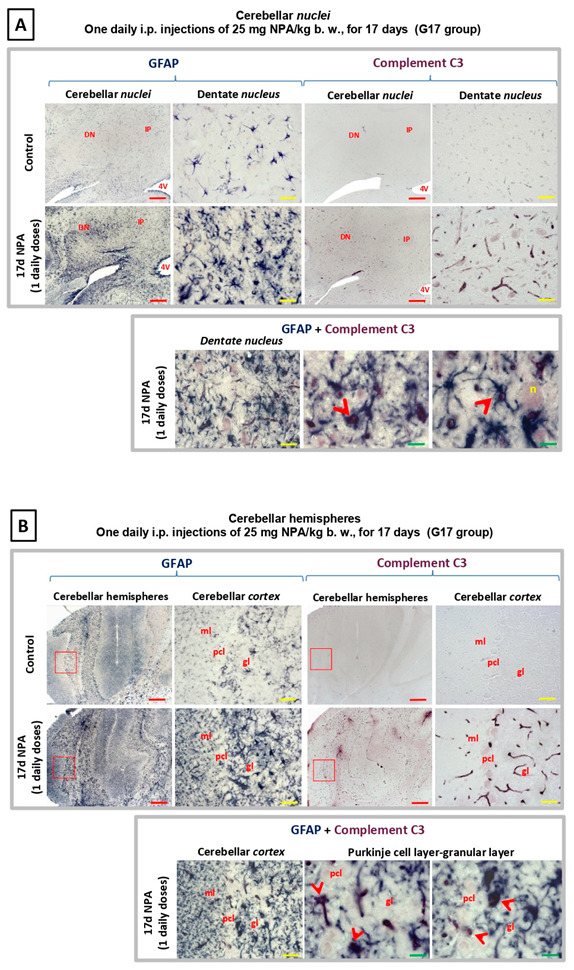
Rats of the G17 group (one daily i.p. injections of 25 mg NPA/kg b. w., for 17 days) show a significant increase of GFAP and Complement C3 in the *cerebellum* with respect to rats of the control group. Light micrographs of coronal sections after immunohistochemistry with anti-GFAP and anti-Complement C3 antibodies. (**A**) Comparative immunolabeling showing an increase in astrocytes expressing C3α and GFAP (upper panel), observed in the cerebellar *nuclei* in experimental rats. C3α is notably detectable at the level of dentate *nucleus*, while it is not observable either in the interposed or the fastigial cerebellar *nuclei*. Note that C3α and GFAP co-localize in the ameboid-shaped astrocytes (arrowhead), detected by using double immunohistochemistry (lower panel). (**B**) Comparative immunolabeling showing an increase in astrocytes expressing C3α and GFAP (upper panel), observed in the cerebellar *cortex* in experimental rats, including the Purkinje and granular layers. Note that C3α and GFAP co-localize in the astrocytes (indicated by red arrowheads), detected by using double immunohistochemistry (lower panel). The red squares indicate the enlarged area shown on respective image of the right side. In red letters: DN: dentate nucleus; IP: interposed nucleus; 4 V: forth ventricle; n: large or projecting neuron; ml: molecular layer; pcl: Purkinje cell layer; gl: granular layer. Red scale bars: 200 µm. Yellow scale bars: 25 µm. Green scale bars: 10 µm.

As shown in Figure 6, an increase in reactive A1 astrocytes expressing C3α and GFAP was also present in the *cerebellum* of rats in G17, revealing the co-location of C3α and GFAP in the ameboid-shaped reactive A1 astrocytes. The analysis of regionalization and distribution of reactive A1 astrocytes showed an increased expression in two specific locations, the cerebellar *nuclei,* and the cerebellar *cortex*. In the first location, C3α is notably detectable at the level of dentate *nucleus*, while it is not observable either in the interposed or the fastigial cerebellar *nuclei* (Figure 6A). In the cerebellar *cortex* (Figure 6B), the increase of C3α and GFAP is especially relevant within the Purkinje and granular layers. This distribution is evident in the most lateral zone of the cerebellar hemispheres and disappears progressively through the midline, being absent in the cerebellar *vermis*. Therefore, there is a significant regionalization of C3 activation that is clearly present in the cerebellar *neocortex*, and not detectable either in the paleo-or the archi-cerebellar *cortex*. Consequently, our data reveal that the corticonuclear lateral band, the most phylogenetically recent cerebellar area, is a highly sensitive cerebellar region to NPA-induced neurotoxicity.

### 2.4. Immunohistochemical Analysis of Regionalization and Location of IL1-α and TNFα Cytokines in the Striatum, Hippocampus, and Cerebellum

**Figure 7:**
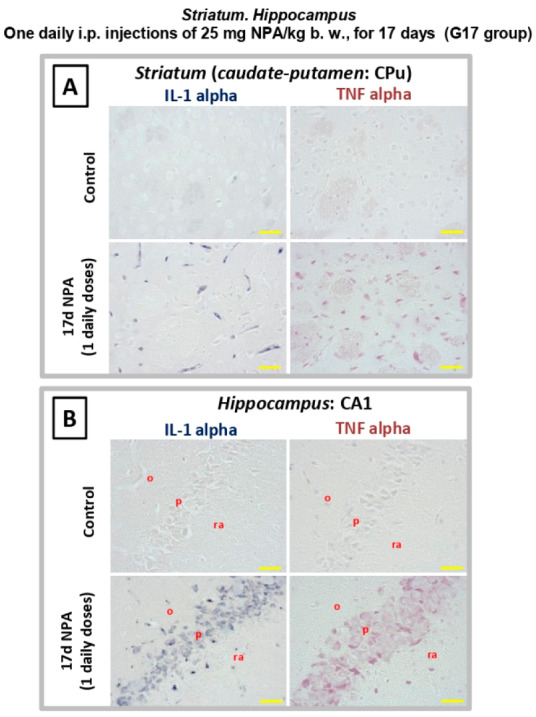
Rats of the G17 group (one daily i.p. injection of 25 mg NPA/kg b. w., for 17 days) show a significant increase of IL1α and TNFα cytokines in the *striatum* and *hippocampus* with respect to rats of the control group. Light micrographs of coronal sections after immunohistochemistry with anti-IL1α and anti-TNFα antibodies. Comparative immunolabeling showing an increase of IL1-α and TNFα expressions in the *striatum* (**A**) and *hippocampus*, within the pyramidal layer and the *stratum radiatum* of CA1 area inside the Ammon’s horn (**B**). In red letters: o: *stratum oriens*; ra: *stratum radiatum*; p: pyramidal layer. Yellow scale bars: 25 µm.

Generation of reactive A1 astrocytes in the brain has been shown to be induced by microglial activation and secretion of IL1α and TNFα cytokines [38]. We experimentally assessed IL1-α and TNFα expressions in the three regions under analysis, that is *striatum*, *hippocampus,* and *cerebellum*. As observed in Figure 7, the treatment with 17 i.p. daily doses of 25 mg NPA/kg (G17) elicits an increase in these cytokines (Figure 7A) within the dorsolateral region of the *striatum* (*caudate* putamen). This increase is also detected within the pyramidal layer and the *stratum radiatum* of CA1 area inside the Ammon’s horn (Figure 7B).

**Figure 8:**
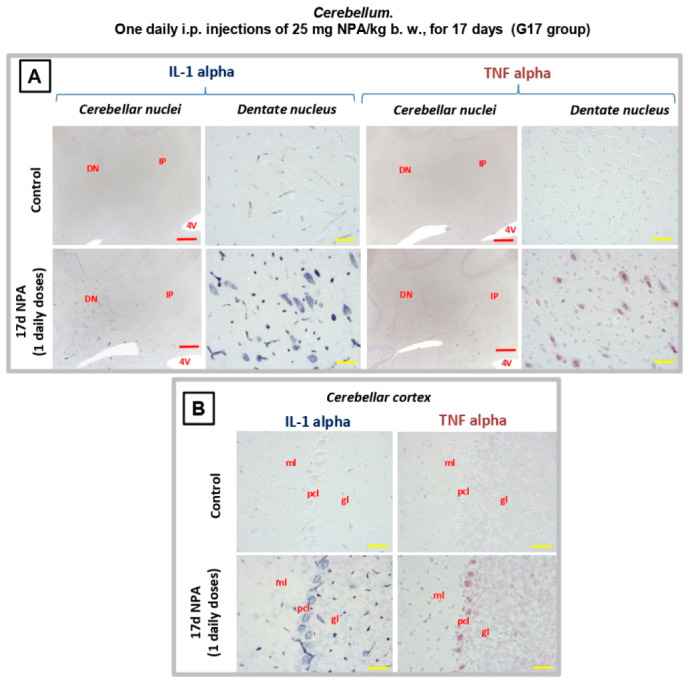
Rats of the G17 group (one daily i.p. injection of 25 mg NPA/kg b. w., for 17 days) show a significant increase of IL1α and TNFα cytokines in the *cerebellum* and with respect to rats of the control group. Light micrographs of coronal sections after immunohistochemistry with anti-IL1α and anti-TNFα antibodies. Comparative immunolabeling showing an increase of IL1-α and TNFα expressions in the *dentate nucleus* (**A**), as well as in the Purkinje and granular layers inside the most lateral zone of the cerebellar cortex (**B**). In red letters: DN: dentate nucleus; IP: interposed nucleus; 4 V: forth ventricle; ml: molecular layer; pcl: Purkinje cell layer; gl: granular layer. Red scale bars: 200 µm. Yellow scale bars: 25 µm.

As observed in Figure 8, cytokines IL1-α and TNFα are also expressed by NPA-activated cerebellar microglia. Our results show their regional distribution in the dentate *nucleus* (Figure 8A), as well as in the Purkinje and granular layers inside the most lateral zone of the cerebellar *cortex* (Figure 8B), highlighting microglial activation in the corticonuclear cerebellar lateral band.

**Figure 9:**
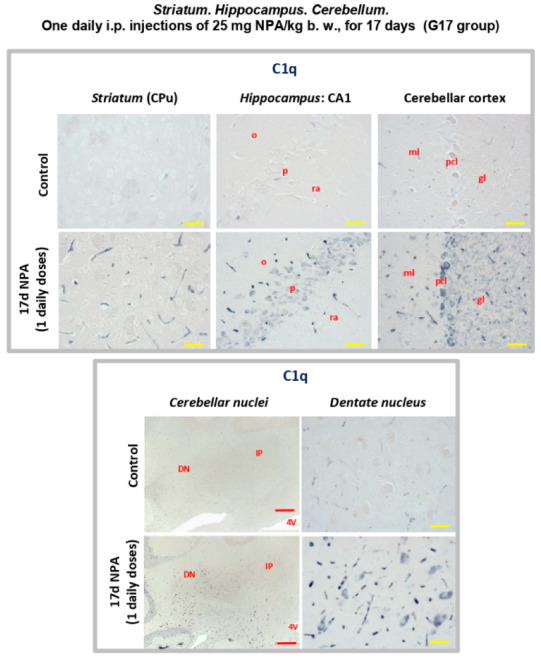
Rats of the G17 group (one daily i.p. injection of 25 mg NPA/kg b. w., for 17 days) show a significant increase of cytokine C1q in the three areas analyzed with respect to rats of the control group. Light micrographs of coronal sections after immunohistochemistry with anti-C1q antibody. Comparative immunolabeling showing an increase of cytokine C1q expression in the *striatum*, *hippocampus* (CA1 area), and cerebellar *cortex* (upper panel), as well as in the dentate nucleus (lower panel). In red letters: o: *stratum oriens*; ra: *stratum radiatum*; p: pyramidal layer; ml: molecular layer; pcl: Purkinje cell layer; gl: granular layer; DN: *dentate nucleus*; IP: interposed *nucleus*; 4 V: forth ventricle. Red scale bars: 200 µm. Yellow scale bars: 25 µm.

Previous authors have reported an increase of cytokine C1q biosynthesis by activated microglia in HD, correlated to an increase of the activation of C3 complement protein [38,41,42]. Our results show a similar distribution of this cytokine in the three regions under analysis (Figure 9) in agreement with the above-mentioned cytokines. Overall, the pattern of increased expression of cytokines (IL1-α, TNFα, and C1q) in *striatum*, *hippocampus,* and *cerebellum* correlated with the regionalization of C3 positive reactive A1 astrocytes.

## 3. Discussion

The results of this work show a significant increase in the expression levels of C3α subunit and cytokines IL-1α, TNFα, and C1q within the *striatum*, *hippocampus,* and *cerebellum*, well before the appearance of the HD-related neurological dysfunctions induced by i.p. NPA injections to adult Wistar rats. Noteworthy, the release of specific cytokines IL-1α, TNFα, and C1q upon microglial activation is required to induce reactive neurotoxic A1 astrocytes expressing C3α subunit [38]. Thus, as a novel finding in NPA-induced neurotoxicity, our results demonstrate an earlier activation of complement C3 protein in the brain from Wistar rats that are treated with this neurotoxin. Other authors have also observed complement C3 expression in astrocytes in the *striatum* obtained from HD post-mortem brain samples [38,41]. However, unlike our observations in initial stages of NPA neurotoxicity, these authors have also described C3 expression in white *matter* and neurons in the *striatum* from the same patients [41], which is possibly well justified due to an advanced lesion stage (from Vonsattel grade 4), when neuronal loss is already evident.

NPA is a mitochondrial toxin that has been shown to produce microglial activation and neuronal death [30], and toxic effects of NPA on astrocytes at early stages of NPA-induced neurodegeneration has been reported [43]. Therefore, our results further validate the use of NPA i.p. administration to shed light on molecular mechanisms in the initial stages of neurotoxicity in HD. Consistently, the astrocytes—which are GFAP positive—present an increased expression of complement C3, suggesting an astrocytosis process characterized by the presence of A1 reactive astrocytes. In the present study, our results show an increase of A1 astrocytes in the three regions under analysis: *striatum*, *hippocampus,* and *cerebellum*. Histochemistry of brain slices in these regions point out that rats treated i.p. with NPA show a marked increase of anti-C3 staining with respect to control untreated rats in the vicinity of or in myelinated axons, striatal spiny neurons, pyramidal neurons of *hippocampus* Ammon’s horn, Purkinje cerebellar neurons, and also in pyramidal neurons of the cerebellar *nuclei*. Since reactive A1 astrocytes are strongly neurotoxic [38], our results strongly support that these neuronal structures are highly vulnerable brain primary targets in NPA-induced neurotoxicity. Interestingly, these neuronal brain structures play a relevant role in motor neurological functions coordinated by the brain, and their degeneration can account for the motor dysfunctions induced by NPA treatment at a later stage. Indeed, kainic acid administration in rats, an animal model of epilepsy neuropathology, also induces a C3 expression increase in the glia of the *hippocampus* as well as in the pyramidal neurons corresponding to CA1 and CA3 areas, neurons particularly sensitive to kainic acid-induced neuronal death [44,45].

It has been proposed that HD is a neurodegenerative disease associated with a neuroinflammatory process mediated by microglial activation in the brain [34,36]. Pro-inflammatory cytokines can promote neuronal death through induction of ROS and also reactive nitrogen species production [29,46,47], excitoxicity caused by the release of L-glutamate [35] and of damage-associated molecular products [47,48], as well as activation and proliferation of astrocytes [47]. It has been reported that the secretion of cytokines IL-1α, TNFα, and C1q upon reactive microglial activation can induce the generation of reactive A1 astrocytes [38]. In a previous work [10] we have shown that there is a large increase of protein nitrotyrosines and depletion of reduced glutathione in NPA-induced degeneration of adult Wistar rat brains, pointing out a large induction of ROS and also reactive nitrogen species in this animal model of HD. In this work we showed that cytokines IL-1α, TNFα, and C1q are also overexpressed in the *striatum*, *hippocampus,* and *cerebellum* of adult Wistar rats treated with daily i.p. injections of 25 mg NPA/kg b.w. In our experimental model, i.p. administration of NPA induced a gradual increase of these three cytokines starting long before the observation of any significant neurological motor impairment in treated rats, indicating an early induction of reactive microglia in the three regions under study (*striatum*, *hippocampus,* and *cerebellum*), and supporting the hypothesis of an early role of the three cytokines in the neuroinflammatory process. Furthermore, the increase of IL-1α, TNFα, and C1q co-localizes with the appearance of C3 positive astrocytes in the three regions. Considering all these data, we conclude that, in our HD experimental animal model, extensive reactive microglial activation takes place in the brain, along with a reactive astrocytosis with generation of neurotoxic A1 astrocytes near or at neuronal structures which play a major role in brain motor coordination. These processes are likely to induce the brain neurodegenerative events that elicit motor disorders observed at an advanced stage of this neurological disease.

Finally, our results provide novel insights into *cerebellum* alterations after NPA administration, which may justify, at least in part, the motor symptoms in these kinds of patients. Previously, it has been reported [16] that NPA administration modifies the levels of metabolite concentrations, specifically of the neuronal activity markers N-acetylaspartate and the osmolyte taurine, in several areas of the rat brain, including the *cerebellum*. Recently, extensive Purkinje cells loss and overall cerebellar damage has been observed in the cerebellar *cortex* from the autopsy of HD cases [18]. As a novel contribution, our results reveal an early expression of inflammatory cytokines and complement C3 at the level of the *dentate nucleus* and the neocerebellar *cortex*. This highlights that NPA administration primarily affects the more recent phylogenetic area of the rat *cerebellum*. Therefore, our experimental NPA model provides an alternate novel tool to study structure–function alterations of the *cerebellum* as a key target in the early stages of HD.

## 4. Materials and Methods

### 4.1. Animals and Treatments

Male Wistar rats, 9–10 weeks old, weighing 290–340 g were housed in a 12 h light/dark cycle and allowed free access to food and water during the experiment. The experimental procedures followed the animal care guidelines of the European Union Council Directive 86/609/EEC. The protocols were approved by the Ethics Committee for Animal Research of the local government.

3-Nitropropionic acid, (>97% by HPLC, Sigma, St. Louis, MO, USA) was administered by intraperitoneal (i.p.) injection of a 20-mg/mL solution in normal saline (0.9% *w*/*v* NaCl). Fresh solutions were prepared by dissolving solid NPA, the pH was adjusted to 7.4 with NaOH 5 M, and filtered through a 0.2 μm filter.

Rats were treated with 25 mg NPA/kg b.w. every 24 h for 6, 17, and 24 days, and are referred in the text as rats from G6 (*n* = 7), G17 (*n* = 7), and G24 (*n* = 7) groups. Of note, the total number of rats used for the G24 group was 10, because three rats died after developing severe pathological symptoms, an interindividual variability that we noticed when adult Wistar rats were treated with doses of 25 mg NPA/kg b.w. every 12 h [10]. Rats from control groups (*n* = 6) received 0.4 mL saline solution (NPA vehicle), with the same treatment schedule given in the experimental groups.

The animals were evaluated for motor impairment throughout the experiment. They were observed daily, just before the injection, and rated with a quantitative scale according to their motor deficiencies [10,49]. This rating scale measures gait abnormalities, hind limbs dystonia, grasping ability, balance, and recumbency.

### 4.2. Preparation of Rat Brain Slices and TTC Staining

The animals were anesthetized with ketamine (50 µg/g), diazepam (2.5 µg/g), and atropine (0.05 µg/g). The brains were immediately removed from the skull and washed in cold phosphate-buffered saline (PBS) pH 7.4, and then cut with a tissue slicer (Stoelting, Woodale, IL, USA).

Some sections (1.5 mm thick coronal slice) corresponding to the three regions analyzed (*striatum*, *hippocampus,* and *cerebellum*) were immersed in a 2% solution of 2,3,5-triphenyltetrazolium chloride (TTC) in PBS for 15 min at 37 °C, and observed under a Leica MZ APO stereomicroscope.

### 4.3. Brain Samples Homogenization and Western Blotting

A group of dissected brain sections of *striatum*, *hippocampus,* and *cerebellum* was weighed and immediately frozen in liquid nitrogen. Thereafter, samples were kept at −80 °C until use.

Brain sections were homogenized within an ice-cold recipient with a glass homogenizer followed by sonication, 30–40 pulses of 1 s, in buffer 25 mM tris-(hydroxymethyl) aminomethane hydrochloride (Tris-HCl) at pH 7.4, 150 mM NaCl, 5 mM ethylenediaminetetraacetic acid, 50 mM NaF, 5 mM NaVO_3_, and 4-(1,1,3,3-Tetramethylbutyl)phenyl-polyethylene glycol (Triton X-100) 0.25%, supplemented with the protease inhibitor cocktail SIGMAFAST S8820 (Sigma-Aldrich). Brain homogenates were centrifuged at 500× *g* for 10 min at 4 °C to pellet tissue debris, the supernatant was carefully removed and supplemented with 40% glycerol, their protein concentration was determined using Bradford’s method, and these samples were conserved at −80 °C until use for Western blotting.

**Figure 10:**
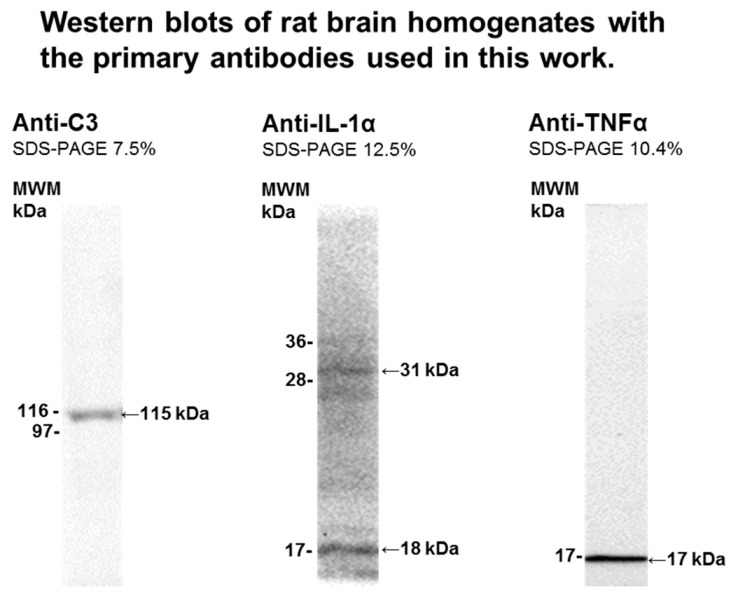
Western blotting of rat brain homogenates with the primary antibodies used in this work. The detection and specificity of primary antibodies for their corresponding target proteins was confirmed by Western blotting: rabbit monoclonal anti-C3 from Abcam (ab200999, dilution 1:2000), mouse monoclonal anti-IL-1α from Santa Cruz Biotechnology (sc-9983, dilution 1:500), and rabbit polyclonal anti-TNFα from Abcam (ab6671, dilution 1:1000). MWM, molecular weight of protein markers closer to the target protein. See Section 4 for further experimental details.

Samples of the homogenates at 1–2 mg of protein/mL were denatured by heating at 98 °C for 5 min in 95 mM Tris-HCl buffer (pH 6.8), 3% sodium dodecyl sulfate (SDS), 1.5% *v*/*v* β-mercaptoethanol, 13% glycerol, and 0.005% bromophenol blue. Then, 20 µg of protein samples were loaded per lane in a polyacrylamide gel of 7.5% or 12% acrylamide, and after running the SDS-polyacrylamide gel electrophoresis the gel was transferred to a polyvinylidene difluoride (PVDF) membrane of 0.2 μm average pore size in standard transfer medium (Trans-BloT TransferMedium, BioRad). PVDF membrane blocking was carried out by 1 h incubation and mild shaking with 3% bovine serum albumin (BSA) in Tris-buffered saline (TBS) supplemented with 0.05% polyoxyethylenesorbitan monolaurate (TBST). Before the incubation with the primary antibody, membranes were washed three times with TBST. The immunodetection of the selected protein (C3, IL-1α, and TNFα) was performed with the following primary antibodies: rabbit monoclonal anti-C3 from Abcam (ab200999, dilution 1:2000), mouse monoclonal anti-IL-1α from Santa Cruz Biotechnology (sc-9983, dilution 1:500) and rabbit polyclonal anti-TNFα from Abcam (ab6671, dilution 1:1000). After incubation with the first antibody overnight or 1 h at room temperature, membranes were washed six times with TBST and incubated for 1 h at room temperature with the appropriate secondary IgG antibody conjugated with horseradish peroxidase. Secondary anti-rabbit IgG-Horseradish peroxidase (Sigma-Aldrich-A0545) or anti-mouse IgG-Horseradish peroxidase (Sigma-Aldrich A0944) were used at a dilution of 1:5000 in TBST. Again, we washed the membrane six times with TBST followed by incubation for 2–3 min with Clarity TM Western ECL Substrate, BIO-RAD. Western blots were revealed with Bio-Rad ChemiDoc^™^ XRS+. The specificity of the detection of primary antibodies was then determined, and membranes were washed with deionized water and treated under continuous stirring at room temperature with the following stripping buffers: (1) 10 min with 0.2 M glycine/0.5 M NaCl brought to pH 2.8 with acetic acid and (2) 10 min with 0.5 M acetic acid/0.5 M NaCl at pH 2.5. After washing with distilled water for 10 min, membranes were blocked with 3% BSA in TBST and treated as indicated above to quantify β-actin to monitor protein load, using mouse monoclonal anti-β-actin (Sigma-Aldrich-A1978, dilution 1:5000) or rabbit polyclonal anti-β-actin (Sigma-Aldrich-A5060, dilution 1:750) as primary antibody, depending on the species of the selected target protein, and the appropriate secondary antibody conjugated with horseradish peroxidase (see above). Figure 10 shows a representative lane of the Western blots obtained with each one of the primary antibodies used for the selected proteins and against β-actin, highlighting the target protein band. Statistical analysis: results of Western blots are expressed as mean ± standard error (s.e.). Statistical analysis was carried out by Mann–Whitney non-parametric test. Significant difference was accepted at the *p* < 0.05 level. All the results were confirmed with triplicate experiments.

### 4.4. Immunohistochemistry

A group of dissected brain sections of *striatum*, *hippocampus,* and *cerebellum* was immersed in 4% paraformaldehyde in PBS, dehydrated in a graded series of ethyl alcohol, cleared in xylene, and embedded in paraffin wax, using standard techniques. Afterwards, tissue blocks were cut in coronal sections (7 µm thick) with a microtome Leica RM2125RT. Slices were hydrated and subjected to immunohistochemistry.

To identify and localize different cells populations we carried out the following procedures.

#### 4.4.1. Glial Fibrillary Acidic Protein (GFAP), Interleukin 1 (IL-1α), and Complement Component 1, Sububcomponent q (C1q-C).

Tissue sections were blocked with 1% BSA in PBS for 30 min, followed by incubation with 5% normal goat serum in 1% BSA and 0.1% Triton X-100 for 2h. Next, slides were incubated overnight at 4 °C in humidified box, with primary antibodies: dilution 1:400 for mouse anti-GFAP (Sigma: G3893), and dilution 1:50 for both mouse anti-IL-1α (Santa Cruz Biotechnology: sc-9983) and mouse anti-C1q-C (Santa Cruz Biotechnology: SC-365301). After extensive washing in PBS, sections were again blocked and the secondary antibody (dilution 1:200) was added, a goat anti-mouse immunoglobulin G conjugated with alkaline phosphatase (IgG-AP), Santa Cruz Biotechnology: sc 3698 for 3 h at room temperature. Finally, the sections were repeated rinsed in PBS, treated with 2 mm levamisole in reaction buffer (100 mm NaCl, 100 mm Tris-HCl pH 9.5, 0.25% polyoxyethylenesorbitan monolaurate, revealed with nitroblue tetrazolium/5-bromo-4-chloro-3-indolyl phosphate (NBT/BCIP) supplied by Roche (catalog n° 1681451) in reaction buffer, washed in PBS, dehydrated and mounted in Eukit.

#### 4.4.2. Complement Component 3, (C3), Tumor Necrosis Factor Alpha (TNFα), and Neurogranin.

Tissue sections were blocked with 1% BSA in PBS for 30 min, followed by incubation with 5% normal goat serum in 1% BSA and 0.1% Triton X-100 for 2 h. Next, slides were blocked with endogenous avidin/biotin blocking kit (Abcam ab 64212) and incubated overnight at 4 °C in humidified box, with primary antibodies: dilution 1:2000 for rabbit anti-C3 (Abcam ab225539), dilution 1:100 for rabbit anti-TNFα (Abcam ab6671), and dilution 1:500 for rabbit anti-neurogranin (Chemicon AB5620). After extensive washing in PBS, endogenous peroxidase activity was quenched with 0.5% H_2_O_2_, again blocked, and incubated with the secondary antibody, a biotinylated goat anti-rabbit immunoglobulin G supplied by Vectastain ABC Kit, Vector Laboratories (PK-6101), for 3 h at room temperature. After rinsing in PBS, the sections were incubated with avidin-biotinylated horseradish peroxidase complex (Vectastain ABC Kit) for 30 min at room temperature. Chromogen development was performed with peroxidase substrate solution (Vector VIP substrate, SK-4600). Slides were washed in distilled water, dehydrated, and mounted in Eukit.

For double immunohistochemistry (GFAP and C3), primary antibodies mouse anti-GFAP and rabbit anti-C3 were applied together and incubated overnight at 4 °C in humidified box. Secondary antibodies, goat anti-mouse conjugated with alkaline phosphatase and biotinylated goat anti-rabbit Vectastain ABC Kit, were applied together, and incubated for 3 h at room temperature. The chromogen development AP anti-GFAP (blue) was developed before POD anti-C3 (red) due to the different pHs used (i.e., develop the more basic pH reaction first to avoid cross-reaction).

#### 4.4.3. Terminal Deoxynucleotidyl Transferase-Mediated Deoxyuridine Triphosphate Nick-End Labelling (TUNEL).

Tissue sections were treated as we have described in a previous publication [50] with an in-situ cell death detection kit, POD (Roche). Apoptotic cells were observed under microscopy using a Vector VIP substrate kit (peroxidase detection; Vector Laboratories).
